# Inter- and intra-reproducibility of genotypes from sheep technical replicates on Illumina and Affymetrix platforms

**DOI:** 10.1186/s12711-016-0267-0

**Published:** 2016-11-10

**Authors:** Donagh P. Berry, Aine O’Brien, Eamonn Wall, Kevin McDermott, Shane Randles, Paul Flynn, Stephen Park, Jenny Grose, Rebecca Weld, Noirin McHugh

**Affiliations:** 1 Animal and Grassland Research and Innovation Centre, Moorepark, Teagasc, Fermoy, Co. Cork Ireland; 2 Sheep Ireland, Highfield House, Shinagh, Bandon, Co. Cork Ireland; 3 Weatherbys Ltd, Naas, Ireland; 4 Identigen Ltd, Dublin, Ireland; 5 GeneSeek, A Neogen Company, Lincoln, NE USA

## Abstract

**Background:**

Accurate genomic analyses are predicated upon access to accurate genotype input data. The objective of this study was to quantify the reproducibility of genotype data that are generated from the same genotype platform and from different genotyping platforms.

**Methods:**

Genotypes based on 51,121 single nucleotide polymorphisms (SNPs) for 84 animals that were each genotyped on Illumina and Affymetrix platforms and for another 25 animals that were each genotyped twice on the same Illumina platform were compared. Genotypes based on 11,323 SNPs for an additional 21 animals that were genotyped on two different Illumina platforms by two different service providers were also compared. Reproducibility of the results was measured as the correlation between allele counts and as genotype and allele concordance rates.

**Results:**

A mean within-animal correlation of 0.9996 was found between allele counts in the 25 duplicate samples that were genotyped on the same Illumina platform and varied from 0.9963 to 1.0000 per animal. The mean (minimum, maximum) genotype and allele concordance rates per animal between the 25 duplicate samples were equal to 0.9996 (0.9968, 1.0000) and 0.9993 (0.9937, 1.0000), respectively. The concordance rate between the two different Illumina platforms was also near 1. A mean within-animal correlation of 0.9738 was found between genotypes that were generated on the Illumina and Affymetrix platforms and varied from 0.9505 to 0.9812 per animal. The mean (minimum, maximum) within-animal genotype and allele concordance rates between the Illumina and Affymetrix platforms were equal to 0.9711 (0.9418, 0.9798) and 0.9845 (0.9695, 0.9889), respectively. The genotype concordance rate across all genotypes increased from 0.9711 to 0.9949 when the SNPs used were restricted to those with three high-resolution genotype clusters which represented 75.2% of the called genotypes.

**Conclusions and implications:**

Our results suggest that, regardless of the genotype platform or service provider, high genotype concordance rates are achieved especially if they are restricted to high-quality extracted DNA and SNPs that result in high-quality genotypes.

## Background

The development of the now commonly termed single nucleotide polymorphism (SNP) chips [1, 2] facilitates the routine generation of genotypes for (hundreds of) thousands of SNPs at a very low cost. There are only a few commercial providers of these SNP chips with most, if not all, studies confined to either Illumina (Illumina Inc, San Diego, CA, USA) or Affymetrix (Affymetrix Inc, San Diego, CA, USA) SNP chips. High concordance rates between genotypes that are generated from both vendors is essential to facilitate switching between platforms; moreover, high reproducibility of genotypes from duplicate biological samples is important for the integrity of downstream statistical analyses.

However, little is known, at least in sheep, on the concordance rate between genotypes that are generated by both platforms on the same animals. In a comparison based on 134 bovine technical replicates, which were both genotyped on the Illumina BovineSNP50 Beadchip, Berry et al. [3] reported mean genotype and allele concordance rates per individual of 0.9989 and 0.9993, respectively. In a comparison between six human technical replicates, Hong et al. [4] documented a mean (standard deviation) genotype concordance rate between Illumina and Affymetrix platforms of 98.80% (0.34%). The objective of our study was to quantify the genotype concordance rate for 84 sheep samples that were each genotyped with a panel of 51,121 SNPs on both an Illumina and an Affymetrix platform. Reproducibility of genotypes that were obtained twice from the same Illumina platform or from two different Illumina platforms was also quantified.

## Methods

DNA was extracted by a single company (Weatherby’s, Ireland) from 89 sheep from multiple breeds and used first to generate genotypes based on 51,135 biallelic SNPs using the commercially available Illumina OvineSNP50 Beadchip (http://www.illumina.com/documents/products/datasheets/datasheet_ovinesnp50.pdf); intensity-only SNPs were not considered in the analysis. Genotyping on the Illumina platform was undertaken by a single commercial company (Weatherby’s, Ireland). Genotype calling was conducted using GenomeStudio Genotyping Module v1.0 (Illumina Dan Diego). The manifest and cluster file were provided by Illumina.

These 51,135 SNPs were then provided to Affymetrix to generate a custom genotyping chip but four of these SNPs were not included on the Affymetrix chip. In addition, no genotypes for 10 of the remaining 51,131 SNPs were generated on the Illumina platform. Thus, none of these 14 SNPs were included in the subsequent analyses. Genotyping on the Affymetrix platform was undertaken by a separate commercial company (Identigen, Ireland) using the previously extracted DNA by Weatherby’s (Ireland) for the Illumina platform. Illumina genotypes were called blind to the genotypes from the Affymetrix platform, and vice versa.

Four and two of the 89 samples that were genotyped on the Affymetrix platform and the Illumina platform, respectively, failed to achieve a 90% call rate (with one of these samples failing to reach the call rate threshold on both platforms). These five samples were not considered further, thus the analysis of the inter-platform reproducibility was based on 51,121 SNPs and 84 individuals.

Separately, 25 animals were genotyped twice on the Illumina OvineSNP50 Beadchip and another 21 samples were genotyped on both the Illumina OvineSNP50 Beadchip and a custom low-density (15,000 SNPs) Illumina Infinium platform that was developed in collaboration with the International Sheep Genomics Consortium. A total of 11,323 SNPs, which were common to both the Illumina OvineSNP50 Beadchip and the custom Illumina platform, were considered in the subsequent reproducibility analysis. The Illumina OvineSNP50 and low-density genotypes were generated using DNA extracted from separate biological samples, i.e., DNA samples used for the Illumina OvineSNP50 platform were extracted and genotyped by Weatherby’s (Ireland) and those used for the Illumina low-density platform were extracted and genotyped by Neogen (GeneSeek, A Neogen Company, Lincoln).

The following statistics were used to compare concordance rates between the duplicate genotypes from the same Illumina platform (n = 25), the genotypes from different Illumina platforms (n = 21), or from the Illumina versus Affymetrix platforms (n = 84): (1) correlation between allele counts; (2) genotype concordance rate defined as average proportion of identical genotypes within SNP or within animal when comparing panels, and (3) allele concordance rate defined as the average proportion of commonly called alleles within SNP or within animal when comparing panels; in this case, a genotype that was called on one platform as heterozygous but homozygous on the other platform was assumed to have one allele in common.

## Results

### Illumina platforms

The mean within-animal correlation between allele counts for the 25 duplicate samples on the Illumina OvineSNP50 platform was 0.9996 and varied from 0.9963 to 1.0000. The within-animal mean (minimum, maximum) allele and genotype concordance rates between these duplicate samples were 0.9996 (0.9968, 1.0000) and 0.9993 (0.9937, 1.0000), respectively. The minimum GC score per duplicate genotype was lower (*P* < 0.001) for discordant genotypes (0.5027) than for concordant genotypes (0.8821). Restricting the comparison to genotypes with a GC score higher than 0.55 improved the mean allele concordance rate across all genotypes from 0.9997 to 0.9999. Re-clustering the genotypes using only the information from the 84 samples from multiple breeds had a minimal effect on the called genotypes; after re-clustering, no homozygous genotype was called as an opposite homozygous and only 3789 of the 4,263,331 called genotypes (i.e., 0.09%) were called as a different genotype (with only one allele different) relative to the genotype called using the Illumina cluster file.

The mean call rate per individual for the same 21 individuals that were genotyped on the two different Illumina platforms was slightly higher (*P* < 0.001) for the Illumina OvineSNP50 platform (0.995) than for the low-density Illumina platform (0.992). Across all SNPs, the mean allele and genotype concordance rates were equal to 0.9997 and 0.9993, respectively. Mean (minimum, maximum) allele and genotype concordance rates per individual were equal to 0.9997 (0.9972, 0.99996) and 0.9993 (0.9954, 0.9999), respectively. A mean (minimum, maximum) within-individual correlation of 0.9994 (0.9954, 0.99993) was found between allele counts. No homozygous genotype on one panel was called as the opposite homozygous on the other panel. The mean GC score for discordant genotypes (0.524) was lower (*P* < 0.001) than that for concordant genotypes (0.863) that were called in both panels.

### Illumina versus Affymetrix platforms

The mean call rate per individual was lower (*P* < 0.001) for the Affymetrix platform (0.974) than for the Illumina platform (0.994). No strong relationship was obvious between individual animal call rates for each platform. On the Illumina platform, 771 SNPs (i.e., 1.51% of all SNPs) had a call rate lower than 0.90, whereas on the Affymetrix platform 4484 SNPs (i.e., 8.78% of all SNPs) had a call rate lower than 0.90; 152 of these SNPs had a call rate lower than 0.90 on both platforms.

**Fig. 1:**
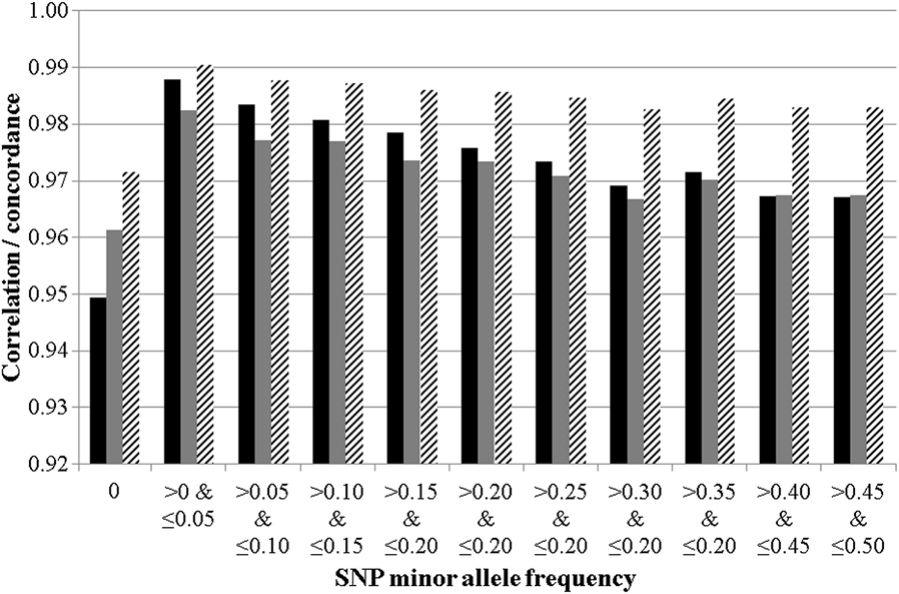
Mean correlation (*black*), genotype concordance rate (*grey*) and allele concordance rate (*striped*) between Illumina and Affymetrix genotypes by single nucleotide polymorphisms (SNP) minor allele frequency from the Illumina platform

**Fig. 2:**
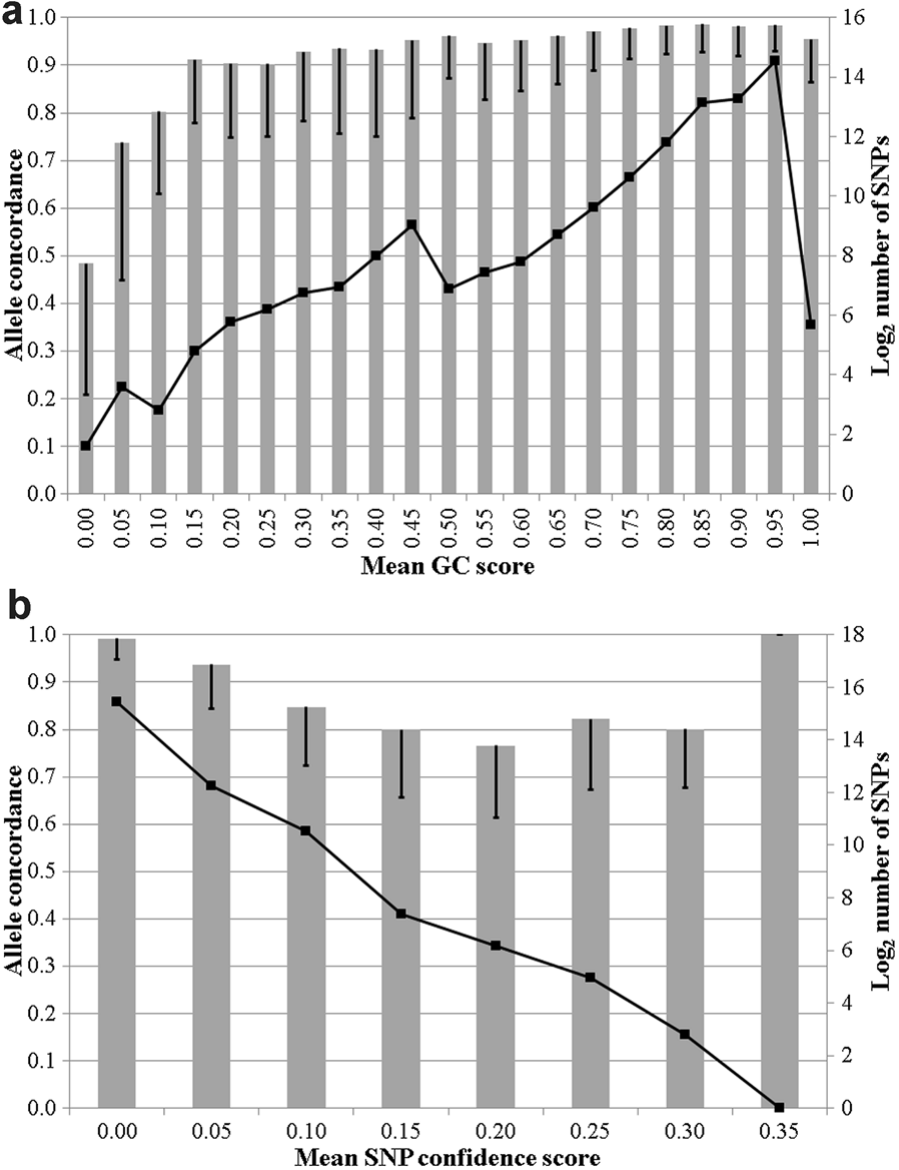
Mean allele concordance rate per single nucleotide polymorphisms (SNP) stratified by Illumina GC score (**a**) and Affymetrix confidence score (**b**) represented by *grey bars* (one standard deviation represented by *standard error bars*) and number of SNPs per score category (*continuous line*). Please note that a higher Illumina GC score but lower Affymetrix confidence score represents superior quality genotypes

Only a very small proportion (i.e., 0.2%) of the homozygous genotypes on one platform were called as opposite homozygous genotypes on the other platform (Table 1). The mean concordance rate per SNP for different minor allele frequency (MAF) bins is in Fig. 1. Concordance rate was best for SNPs with a MAF between 0 and 0.10 and worst for monomorphic SNPs. Concordance rate decreased as the mean SNP quality score decreased (Fig. 1), which is represented as a lower GC score for Illumina genotypes and a higher confidence score for Affymetrix genotypes. Of the 160 SNPs that had an allele concordance rate between both panels lower than 0.50, 60 (i.e., 37.5%) had a mean GC score lower than 0.55, which is the threshold commonly used in association studies (Fig. 2). The mean GC score was lower (*P* < 0.001) for discordant genotypes (0.8549) than for concordant genotypes (0.8894).

**Table 1:** Contingency table of the genotypes (0 = AA, 1 = AB, 2 = BB) from Illumina and Affymetrix platforms for all 51,121 SNPs on 84 animals

Illumina	Affymetrix		
	0	1	2
0	97.4	2.24	0.34
1	2.13	95.34	2.53
2	0.33	1.36	98.31

Excluding the SNPs with an allele concordance rate between panels lower than 0.80, the mean genotype (allele) concordance rate per SNP for the remaining 49,859 SNPs was 0.9805 (0.9902). The mean concordance per SNP categorised into the different SNP categories assigned by Affymetrix is in Table 2. Mean call rates were highest for the SNPs that were categorised by Affymetrix as having high resolution clusters.

**Table 2:** Number of SNPs (N) and mean correlation, genotype concordance rate, allele concordance rate for each SNP category designated by Affymetrix

Category	N	Correlation	Genotype concordance rate	Allele concordance rate
PolyHighResolution	37,619	0.9931	0.9913	0.9956
NoMinorHom	2135	0.9912	0.9882	0.9933
Monohigh	972	0.9188	0.9336	0.9518
CallRateBelowThres	4288	0.9624	0.9513	0.9752
OffTargetVariant	463	0.8215	0.6951	0.8454
Other	5641	0.8313	0.8024	0.8933

The mean within-animal correlation between genotypes that were generated on the Illumina and Affymetrix platforms was equal to 0.9738 and varied from 0.9505 to 0.9812. The mean (minimum, maximum) within-animal genotype and allele concordance rates were 0.9711 (0.9418, 0.9798) and 0.9845 (0.9695, 0.9889), respectively.

## Discussion

Inaccurate or low reproducibility genotypes have repercussions on genomic predictions [5], genome-wide association studies [4] and other analyses such as parentage verification and assignment as well as estimation of coancestry. The ability to readily switch between providers of genotyping technologies, without impacting the integrity of the data after being collated, can contribute to put greater pressure on vendors to reduce genotyping costs further.

### Reproducibility of the Illumina panel

The mean within-animal genotype and allele concordance rates of 0.9993 and 0.9996, respectively between duplicate samples on the Illumina OvineSNP50 platform and also the near unity genotype and allele concordance rates of 0.9993 and 0.9997, respectively between the two Illumina platforms, are excellent and corroborate the respective values of 0.9989 and 0.9993 reported by Berry et al. [3] using duplicate genotypes of 134 cattle that were genotyped on the Illumina BovineSNP50 beadchip. Using six samples from the human HapMap project, Hong et al. [4] reported a mean genotype concordance rate of 0.9940 between duplicate samples on an Illumina platform and of 0.9987 between duplicate samples on an Affymetrix platform; all genotype comparisons undertaken by Hong et al. [4] originated from the same genotyping laboratory.

The range in mean concordance rate per individual genotyped in our study on the same Illumina platform or different Illumina platforms was also minimal, which suggests consistently excellent reproducibility. Although the retrospective nature of the analyses undertaken in the present study did not make it possible to disentangle various effects of the genotyping laboratory, DNA extraction method, or Illumina platform used, the fact that the concordance rate was excellent between genotypes that were generated by two different genotyping laboratories on two different Illumina platforms from DNA extracted by two separate laboratories suggests that all three factors actually have a minimal effect on the generated genotypes. However, only two (experienced) laboratories were compared, which limited the possibility that a laboratory effect impacted genotype. Nonetheless, the high concordance rate between duplicate bovine samples reported by Berry et al. [3], based on genotypes that were generated across multiple laboratories, provides further confidence that there is good genotype concordance across different service providers. Furthermore, most, if not all, of the discrepancies between duplicate genotypes on the platforms used in our study were actually due to a homozygous genotype of one replicate being called as a heterozygous in the other replicate, or vice versa. Moreover, applying stricter quality control on the GC score of the called genotype could improve the reliability of the genotype furthermore; a default threshold GC score of 0.15 is applied in GenomeStudio, thus genotypes with a GC score lower than 0.15 are not called by default. However, our results suggest that a more stringent threshold should be imposed, possibly higher than 0.50 (Fig. 2). This has already been done in some studies in which, only genotypes that had a GC score higher than 0.60 were retained [6].

### Reproducibility between panels

The high concordance rate between genotypes that were generated on the two different platforms is consistent with documented reports from human studies that used either six duplicate samples (genotype concordance rate of 0.9880; see [4]) or 396 duplicate samples (genotype concordance rate of 0.9989; see [7]) that were genotyped on both Illumina (Infinium array) or Affymetrix platforms. However, Jiang et al. [7] undertook their concordance analysis after quality control of the genotypes, which involved the exclusion of SNPs with a low (i.e., <0.01) MAF and poor (<0.95) call rate, which left only 62.28% of the SNPs on the original Affymetrix platform and 57.04% of SNPs on the original Illumina Infinium platform.

Affymetrix probe sets are classified into six categories (Table 2) by the Affymetrix Axiom software based on quality control metrics; the SNP clustering properties for each of the six categories is graphically illustrated in Liu et al. [8]. A SNP is (1) “PolyHighResolution” if three good resolution clusters (i.e., homozygous wild, heterozygous, homozygous individuals) are formed; (2) “NoMinorHom” if only two clusters are formed with no genotype for one homozygous individual; (3) “MonoHighResolution” if the called genotypes are all monomorphic; (4) “Off-target variants”, if three clusters are formed, but with one additional off-target cluster due to sequence dissimilarity between the probes and the target genome regions; (5) “CallRateBelowThreshold” if the call rate of the SNP is below the call rate threshold but the cluster properties are above the threshold, and (6) “Other” if more than one of the cluster properties are below the threshold. Thus, the fact that the concordance rate was higher for SNPs that are defined as “PolyHighResolution” is not unexpected but this category of SNPs represented only 73.5% of all SNPs. However, genotyping a larger number of animals may increase the likelihood of identifying more genotypic variability and could therefore contribute to the clustering property of SNPs classified in our study as “NoMinorHom” being changed to “PolyHighResolution”; “NoMinorHom” SNPs represented 4.2% of the data. Therefore, restricting SNPs to those classified as high-quality is likely to improve the reliability of the genotypes. Furthermore, most studies impose a restriction on the MAF of SNPs prior to inclusion in analyses; concordance rate of monomorphic SNPs was poorer than that of all other SNPs (Fig. 1). Restricting the SNPs to only the segregating “PolyHighResolution” SNPs increased the mean genotype concordance rate across all SNPs from 0.9712 to 0.9949.

In conclusion, our findings indicate that genotype data obtained from the panels investigated here can be readily combined with little expected loss in the integrity of subsequent analyses especially if quality control measures are imposed. However it was not feasible in this retrospective analysis to actually determine the truly correct genotype, thus we cannot make any inference as to which platform was most accurate. It should also be noted that our results are based on high-quality DNA samples using standard DNA extraction methods; thus, we cannot draw conclusions on the absence of discrepancies if lower quality DNA samples (e.g., from embryo biopsies or from high-throughput DNA extraction methods) are used.
